# Neutrophil-Associated Inflammatory Changes in the Pre-Diabetic Pancreas of Early-Age NOD Mice

**DOI:** 10.3389/fendo.2021.565981

**Published:** 2021-03-10

**Authors:** Yesica Garciafigueroa, Brett E. Phillips, Carl Engman, Massimo Trucco, Nick Giannoukakis

**Affiliations:** ^1^ Institute of Cellular Therapeutics, Allegheny Health Network, Pittsburgh, PA, United States; ^2^ Department of Biological Sciences, Carnegie Mellon University, Pittsburgh, PA, United States

**Keywords:** neutrophils, type 1 diabetes, autoimmunity, neutrophil elastase, myeloperoxidase, non-obese diabetes (NOD) mice, AZD5904, AZD9668

## Abstract

A growing body of evidence indicates that neutrophils are the first major leukocyte population accumulating inside the pancreas even before the onset of a lymphocytic-driven impairment of functional beta cells in type 1 diabetes mellitus (T1D). In humans, pancreata from T1D deceased donors exhibit significant neutrophil accumulation. We present a time course of previously unknown inflammatory changes that accompany neutrophil and neutrophil elastase accumulation in the pancreas of the non-obese diabetic (NOD) mouse strain as early as 2 weeks of age. We confirm earlier findings in NOD mice that neutrophils accumulate as early as 2 weeks of age. We also observe a concurrent increase in the expression of neutrophil elastase in this time period. We also detect components of neutrophil extracellular traps (NET) mainly in the exocrine tissue of the pancreas during this time as well as markers of vascular pathology as early as 2 weeks of age. Age- and sex-matched C57BL/6 mice do not exhibit these features inside the pancreas. When we treated NOD mice with inhibitors of myeloperoxidase and neutrophil elastase, two key effectors of activated neutrophil activity, alone or in combination, we were unable to prevent the progression to hyperglycemia in any manner different from untreated control mice. Our data confirm and add to the body of evidence demonstrating neutrophil accumulation inside the pancreas of mice genetically susceptible to T1D and also offer novel insights into additional pathologic mechanisms involving the pancreatic vasculature that have, until now, not been discovered inside the pancreata of these mice. However, inhibition of key neutrophil enzymes expressed in activated neutrophils could not prevent diabetes. These findings add to the body of data supporting a role for neutrophils in the establishment of early pathology inside the pancreas, independently of, and earlier from the time at onset of lymphocytic infiltration. However, they also suggest that inhibition of neutrophils alone, acting *via* myeloperoxidase and neutrophil elastase only, in the absence of other other effector cells, is insufficient to alter the natural course of autoimmune diabetes, at least in the NOD model of the disease.

## Introduction

Type 1 diabetes mellitus (T1D) is a multifaceted chronic and progressive autoimmune syndrome that results in the functional impairment as well as the physical eradication of pancreatic insulin-producing beta cells (1, 2). Although the main effectors of beta cell impairment and death are T-cells, leukocytes of the innate arm of the immune system are active in the early part of the inflammation process inside the pancreas. In rodent models of T1D, especially the non-obese diabetic (NOD) strain, pancreas-resident macrophages and dendritic cells are activated many weeks prior to the onset of lymphocyte accumulation (3–7) in response to an as-yet unidentified intrapancreatic microanatomical pathology. Recent evidence, however, also shows that the pathology may have its basis in the non-endocrine regions of the pancreas and that this pathology is first encountered by resident and subsequently migratory leukocytes of the innate arm of the immune system (2, 8–12).

Neutrophils are possibly the most powerful leukocytes of the innate arm of the immune system in terms of responses to damage and danger signals. Together with mast cells, they act as the first responders to microenvironmental damage, and when uncontrolled, activated neutrophils establish and exacerbate a process of sterile inflammation (9, 13–19). While neutrophil accumulation early in the inflammation of the pancreas in rodent models of T1D was observed more than 2 decades ago (18–22), their more precise contribution was established in the elegant studies of Diana and colleagues (23) in the non-obese diabetic (NOD) mouse strain, a well-established rodent model of spontaneous and progressive autoimmune T1D (although this strain also exhibits autoimmunity to other organs including the thyroid and the salivary gland) (24, 25). Neutrophil infiltration and neutrophil extracellular trap [NET (26, 27)] formation in the islets were observed as early as 2 weeks after birth, and blockage of neutrophil activities with an anti-Ly6G antibody reduced the subsequent development of insulitis and diabetes (23). Furthermore, self-DNA:anti-DNA IgG immune complex formation inside the pancreas triggered the accumulation of neutrophils as well as the production of NETs and the release of the cathelicidin peptide CRAMP. These complexes activated plasmacytoid dendritic cells to release IFNα, which is believed to participate in the onset of accumulation of autoreactive T-ells into the pancreas (23). Further studies showed that pancreatic macrophages and β cells which produce chemokines CXCL1 and CXCL2 can recruit CXCR2‐expressing neutrophils to the pancreatic islets from the circulation (28).

Neutrophils are the most abundant leukocyte population in humans, constituting between 40 and 75% of the total leukocyte population. In humans, they are now confirmed to be involved in the onset and progression of T1D (29, 30). Notably, circulating neutrophil counts are reduced in patients with T1D and in their non-diabetic first-degree relatives (30). The number of circulating neutrophils was shown to be reduced in patients with T1D as well as in presymptomatic at-risk subjects (29–33). Battaglia and colleagues further reported that neutrophils infiltrate the pancreas of donors with T1D while they are absent in the pancreata of non-diabetic or type 2 diabetic donors (30), although others have observed neutrophil accumulation inside the pancreata of type 2 donors (34, 35). Importantly, histological analysis of pancreata from four different international cohorts revealed neutrophil accumulation as well as NET formation in T1D donors at all disease stages, including in T1D autoantibody-positive subjects at risk of the disease (10, 29, 31–33). The observations of NET formation indicates that neutrophils are directly pathogenic in the pancreas of humans who progress to T1D, consistent with observations made in the NOD mouse strain (23).

Neutrophil serine proteases, including neutrophil elastase (NE), proteinase 3 (PR3), and cathepsin G (CG), are the major components of neutrophil azurophilic granules that participate in the elimination of engulfed microorganisms (36, 37). Neutrophil activation and degranulation can result in the release of neutrophil serine proteases into the extracellular medium and circulation, where they not only help to eliminate the invaded pathogens but also serve as the humoral regulators of the immune responses during acute and chronic infl ammation, modulating cellular signaling network by processing chemokines, and activating specific cell-surface receptors (38–40). Abnormal activities of neutrophil serine proteases have been implicated in the pathogenesis of several inflammatory and autoimmune diseases, including chronic obstructive pulmonary disease, cystic fibrosis, Wegener granulomatosis, Papillon-Lefèvre syndrome, and small-vessel vasculitis (36). Wang et al. showed that circulating neutrophil serine proteases including NE and PR3 increased significantly in patients with T1D and were closely associated with β cell autoimmunity, suggesting a role of neutrophil activation in the onset and pathogenesis of the disease (33). In that study, these investigators also demonstrated that alpha-1 antitrypsin, a natural inhibitor of NE, was associated with reduced neutrophil counts in patients with T1D. Furthermore, elevated NE and PR3 were significantly associated with the positive numbers and titers of the autoantibodies detected in T1D patients (33). In the NOD mouse, these authors also discovered that elevated circulating NE/PR3 activities occur well before the onset of hyperglycemia and diabetes and that their activities gradually decline after the development of overt diabetes.

Based on these observations, we proposed that a more targeted approach to preventing the detrimental effects of neutrophils inside the pancreas would prevent the onset and progression of type 1 diabetes without resorting to neutrophil depletion or using approaches with significant off-target effects. Towards this objective, we considered the use of pharmacologic inhibitors of NE and MPO, alone or in combination, to delay and/or prevent the progression of T1D in the NOD mouse strain. In these studies, we confirmed the observations that neutrophils accumulate as early as 2 weeks of age inside the pancreas of NOD mice but we also made the following notable and novel observations until now unknown, or at least unreported. First, we observed the expression of NET components in very early age in NOD mice. These components (NE and citrullinated histone H3; CitH3) were broadly distributed inside the pancreatic parenchyma. This was preceded by a very dense accumulation of what are very likely platelets surrounding pancreatic vessels together with extravascular von Willebrand factor (vWF). These observations were not seen in C57BL/6 mice. Our data indicate, for the first time, that the NOD mouse strain exhibits vascular pathology almost immediately after birth and that this is associated with the concomitant temporal accumulation of neutrophils, followed soon by the expression of NET components. However, and contrary to our expectations, based on these observations as well as those by others, we also discovered that pharmacologic inhibitors of NE [AZD9668 (41)] and MPO [AZD5904 (42)], alone or in combination, were ineffective in delaying and/or preventing the progression of T1D towards overt hyperglycemia.

## Materials and Methods

### Animals

Male and female NODLt/J and C57BL/6J mice between the ages of 2 and 10 weeks were purchased from the Jackson Laboratories (Bar Harbor, ME, USA) and maintained in a specific pathogen-free environment at the Animal Research Facility of the Allegheny Health Network. All mice were 2 weeks of age upon arrival at our facility. Where the effect of study agents (MPO/neutrophil elastase inhibitors) on diabetes incidence was the primary outcome measure in T1D diabetes prevention, mice were randomized into the treatment arms and study agents were administered thereafter, initiated at 3 weeks of age. All experiments and animal euthanasia were conducted in line with specific protocols approved by the Allegheny Health Network IACUC.

### Immunofluorescence Microscopy

Following euthanasia, pancreata were excised and placed inside 2.5% paraformaldehyde (Sigma-Aldrich, MO, USA) for 3–4 hrs. Pancreata were transferred to 30% sucrose (Sigma-Aldrich, MO, USA) overnight and then embedded in NEG-50 (Fisher Chemicals, NJ, USA). Then 5–10 micron frozen serial sections were subsequently cut. The sections were permeabilized and blocked with 20% Normal Goat Serum for 1 h at room temperature prior to staining with fluorochrome-conjugated antibodies (single or in pairs). The following antibody clones were used: rat anti-Ly6G (Abcam, ab25377, 1:100, clone RB6-8C5), rabbit anti-NE (Abcam, ab21595, 1:100), mouse anti-CD31 (Abcam, ab24590, 1:100, clone P2B1), guinea pig anti-insulin (Abcam, ab7842, 1:100), rabbit anti-citrullinated histone H3 (Abcam, ab5103, 1:250), sheep anti-vWF (Abcma, ab11713, 1:100), mouse anti-PSGL-1 (Abcam, ab78188, 1:100, clone KPL-1), rat-anti CD42b (Emfret Analytics Germany, #M040-0, 1:100, clone Xia.G5). Immunofluorescence images were obtained using a Zeiss Axio Observer Z1 inverted fluorescence microscope with the Zen 2012 Blue Edition software. The number of cells positive for Ly6G, NE, anti-citrullinated histone H3, and DAPI nuclear stain were determined using the QuPath Software version 0.1.2 (43). Averages were determined from quantification in three to five slides per animal per antibody target and presented graphically as the number of marker-positive cells per 100 total cells.

### Pharmacologic MPO and NE Inhibitors

AZD5904 (a specific MPO inhibitor) (42) and AZD9668 (a specific neutrophil elastase inhibitor) (41) were provided by Astra Zeneca under a collaborative research agreement supported by the NIH National Center for Advancing Translational Sciences (NCATS). These agents were manufactured into rodent chow (Purina LabDiet 5053 ground and repelleted with study agents; Research Diets, New Brunswick, NJ, USA) for oral drug delivery. Study diets were produced that contained either 312.50 mg AZD5904/kg diet, 83.33 mg/g AZD9668, or a combined 312.50 mg AZD5094+83.33 mg AZD9668/kg diet, or no-drug diet and were administered *ad libitum* resulting in an expected steady state dose of 75 mg/kg body weight (for AZD5904) (42) and 10 mg/kg/body weight (for AZD9668) (41). Depending on the experiment, mice were placed on diets manufactured with one or both of the inhibitors, or control diet, beginning at 3 weeks of age and were maintained on these diets until the time of euthanasia.

### Diabetes Ascertainment in NOD Mice *In Vivo*

Female NOD mice were randomly assigned into four diet cohorts (no drug, AZD5904 alone, AZD9668 alone, AZD5904+AZD9668; n = 8–10 mice per arm) and fed the diets *ad libitum* beginning at 3 weeks of age. They were assessed for blood glucose levels beginning at 10 weeks of age using a OneTouch Ultra glucometer (Lifescan, Malvern, PA, USA). Blood glucose was measured twice weekly to assess diabetes onset. Diabetes was defined as two consecutive readings, spaced 1 day apart, of >300 mg/dl. Mice that developed diabetes were euthanized within 2 weeks of diabetes onset. Mice that remained diabetes-free were maintained on study diets up to 35 weeks of age at which time they were euthanized.

### Pancreatic and Spleen MPO and NE Enzyme Activity *In Vitro*

Resected pancreatic tissue was dissociated into single cells using the gentleMACS Octo Dissociator with Heaters (Miltenyi Biotec) utilizing a custom program. The solution the tissues were dissociated in was 0.5 ml of MPO assay buffer from the MPO Colorimetric Activity Assay Kit (Sigma-Aldrich). Samples were centrifuged to remove cells and debris and the clarified supernatant was assessed in the enzyme assay following the manufacturer’s instructions. The same supernatant was used to measure neutrophil elastase activity using a commercially-available kit (Cayman Chemical). Supernatant protein concentration was measured by BCA assay (Peirce). Data are reported as units of activity per mg of protein.

### Statistical Analyses

We tested for any significance in the time at onset of, and progression to diabetes (onset of hyperglycemia and rate of onset of hyperglycemia) among the study arms using the log-rank test for survival. Student’s t-test was used to test for signifance in cell population differences in microscopy sections on a-per week of age basis, as well as for comparing the quality of freshly isolated cell suspensions. Student’s t-test was also used to determine statistical significance in enzyme activity *in vitro*. All statistical analyses were conducted using GraphPad Prism version 7 software. A p value of <0.05 was considered to indicate statistical relevance to the differences in the outcomes in all statistical tests listed.

## Results

### Neutrophil Accumulation Is Evident as Early as 2 Weeks of Age in the Pancreas of NOD Mice

**Figure 1:**
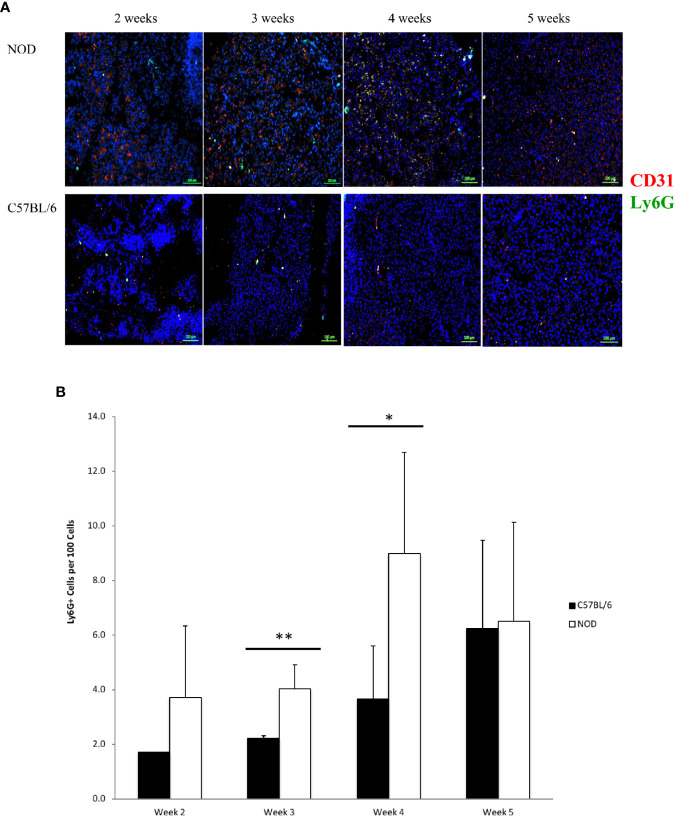
Post-natal pancreas from NOD female mice exhibit Ly6G and CD31 events. **(A)** Serial sections from formalin-fixed paraffin-embedded pancreata of 2-5 week old female NOD and C57BL/6 mice were stained with fluorochrome-tagged antibodies to Ly6G and CD31. Immunofluorescence shown is representative of outcomes observed in at least three individual mice of each strain. Bar at the bottom of each image represents the length of 100 μm. **(B)** Quantification of Ly6G+ signals in serial sections from post-natal pancreas of NOD female and C57BL/6 female mice. The number of Ly6G events and DAPI nuclear stain were determined using the QuPath Software version 0.1.2. Means of positive signals, following autofluorescence removal, were determined among three to five slides per pancreas per antibody target and presented graphically as the number of marker-positive cells per 100 total cells. Using Ly6G+ as a marker of neutrophils, their accumulation was evident at 4 weeks of age in the pancreas of NOD mice. By 5 weeks of age, most of the Ly6G+ signal was found at lower frequency. The number of Ly6G+ events is elevated in NOD, reaching significance at 3 and 4 weeks of age. Statistical significance was determined by a student’s t-test at individual weeks of age with a of p < 0.05 designated by * and p < 0.01 designated by **.

Using immunofluorescence microscopy to determine the presence and accumulation of neutrophils inside the pancreas from the youngest possible age we could formally euthanize under our IACUC permissions, we observed evidence of neutrophils and neutrophil activity at the earliest possible age of 2 weeks from birth. In serial sections from individual mice, we consistently observed Ly6G+ signals representative of neutrophils (**Figure 1A**). Using Ly6G+ as a marker of neutrophils, their accumulation was evident at 4 weeks of age in the pancreas of NOD mice. By 5 weeks of age, most of the Ly6G+ signal was found at lower frequency even though the differences did not reach statistical significance (**Figure 1B**). In all instances of Ly6G+ signals, they were distributed in a random manner in the non-endocrine tissue of the pancreas.

In order to determine events related to possible neutrophil clearance, we monitored the onset of CD31+ signals [although CD31 can also represent endothelial cells (44–47)]. CD31 positivity was observed at 2 weeks of age and persisted up to the end of our monitoring period (5 weeks). At 4 weeks of age, CD31 and Ly6G were, qualitatively, mostly co-localized. In contrast to Ly6G+ and CD31+ events in all the serial sections from pancreata of all NOD mice, few of all serial sections from all C57BL/6 mice (n = 5 sections from an n = 1–2 mice depending on age) exhibited Ly6G+ or CD31+ signal. Their distribution, where observable, was also random inside the non-endocrine tissue of the pancreas (**Figure 1A**).

### Citrullinated Histone H3 Expression Is Evident at 4 Weeks of Age and Is Widely Distributed in the Pancreas of NOD Mice

**Figure 2:**
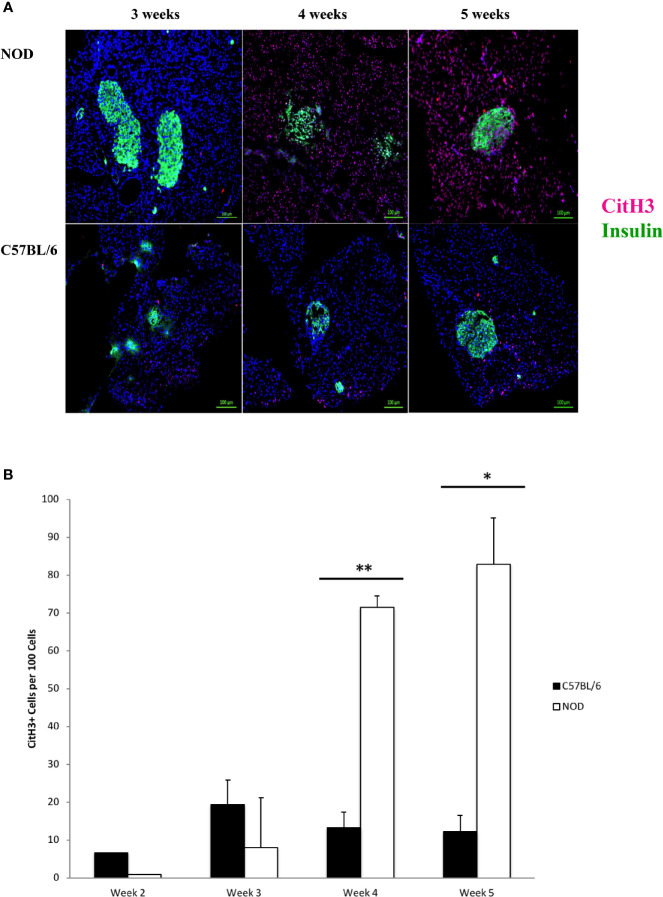
Generalized distribution of citrullinated histone H3 in mostly the non-endocrine tissue of NOD, but not C57BL/6 pancreata beginning at 4 weeks of age. **(A)** Citrullinated histone H3 is considered to be an informative marker of neutrophil extracellular trap formation. Serial sections from formalin-fixed paraffin-embedded pancreata of 3-5 week old female NOD and C57BL/6 mice were stained with fluorochrome-tagged antibodies to insulin and citrullinated histone H3 (CitH3). Immunofluorescence shown is representative of outcomes observed in at least three individual mice of each strain. Bar at the bottom of each image represents the length of 100 μm. **(B)** Quantification of CitH3+ signals in serial sections from post-natal pancreas of NOD female and C57BL/6 female mice. The number of CitH3+ events and DAPI nuclear stain were determined using the QuPath Software version 0.1.2. Means of positive signals, following autofluorescence removal, were determined among three to five slides per pancreas per antibody target and presented graphically as the number of marker-positive cells per 100 total cells. The number of CitH3+ events is elevated in NOD, reaching significance at 4 and 5 weeks of age. Statistical significance was determined by a student’s t-test at individual weeks of age with a of p < 0.05 designated by * and p < 0.01 designated by **.

We hypothesized that the accumulation of neutrophils inside the pancreas before 5 weeks of age, and potential neutrophil clearance as early as 2 weeks of age represented a response to a pathologic condition. This potential pathologic response could trigger the formation of neutrophil extracellular traps, or neutrophil extracellular traps could underly the possible presence of CD31+ in the pancreata of NOD mice, but not C57BL/6 mice. Citrullinated histone H3 has been shown to co-localize with neutrophil extracellular traps and is considered to be a reliable marker of NET formation (48–60). We therefore used this marker as a surrogate of possible NET formation in the pancreata of NOD mice. In **Figure 2A** we show a remarkable accumulation of citrullinated histone H3 beginning at 4 weeks of age. Like Ly6G+ signals, citrullinated histone H3 is distributed broadly and randomly throughout mostly the non-endocrine tissue although in NOD mice who are 4 and 5 weeks of age. We note an intra-islet distribution of citrullinated histone H3 that appears to co-incide with insulin positivity (**Figure 2A**). It was unclear if this localization with insulin represents beta cells undergoing citrullination of histone H3 or formation of neutrophil extracellular traps at or physically close to beta cells. When we examined age- and sex-matched pancreata from C57BL/6 mice for citrullinated histone H3 we did not discern any significant accumulation until 5 weeks of age (**Figure 2**). Quantitation of citrullinated histone H3 signals in serial sections from 2 to 5 week old mouse pancreata identified statistically-distinguishable differences between the means of NOD and C57BL/6 mouse pancreata at 4 and 5 weeks of age, but not at 2 or 3 weeks (**Figure 2B**). In the same serial sections, the mass of insulin-postivie tissue, representative of islets, was comparable. These data suggested that the neutrophils accumulated inside the pancreas of NOD mice as early as 2 weeks of age could be activated and therefore should express neutrophil elastase.

**Figure 3:**
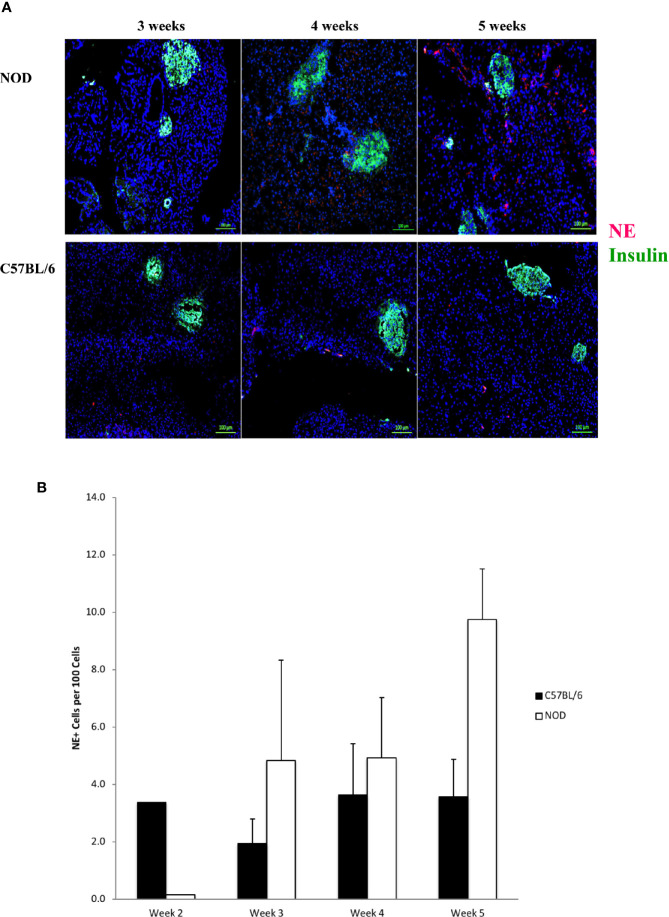
Neutrophil elastase positivity is evident at 4 weeks of age but does not co-localize within islets. **(A)** Serial sections from formalin-fixed paraffin-embedded pancreata of 3-5 week old female NOD and C57BL/6 mice were stained with fluorochrome-tagged antibodies to insulin and neutrophil elastase (NE). Immunofluorescence shown is representative of outcomes observed in at least three individual mice of each strain. Bar at the bottom of each image represents the length of 100 μm. **(B)** Quantification of NE+ signals in serial sections from post-natal pancreas of NOD female and C57BL/6 female mice. The number of NE+ events and DAPI nuclear stain were determined using the QuPath Software version 0.1.2. Means of positive signals, following autofluorescence removal, were determined among three to five slides per pancreas per antibody target and presented graphically as the number of marker-positive cells per 100 total cells. The differences among ages did not reach statistical significance by ANOVA and neither did difference between NOD and C57BL/6 mice at specific ages (Student’s t-test).

In **Figure 3A** we show that neutrophil elastase is detectable, exhibiting a random extra-islet distribution, at 4 and 5 weeks of age in NOD mice, concurrent with the age at onset of Ly6+ accumulation and the presence of citrullinated H3 histone. Neutrophil elastase was not detected to any significant level in the pancreata of C57BL/6 mice of any age (**Figure 3A**). Quantitation of NE signals in serial sections from 2 to 5 week old mouse pancreata identified statistically-distinguishable differences between the means of NOD and C57BL/6 mouse pancreata at only 5 weeks of age, although the differences between the means at the other ages exhibited a trend favoring higher signal in NOD mice compared to C57BL/6 (**Figure 3B**).

### Vascular Immunopathology Is Evident as Early as 2 Weeks of Age in the Pancreas of NOD Mice

**Figure 4:**
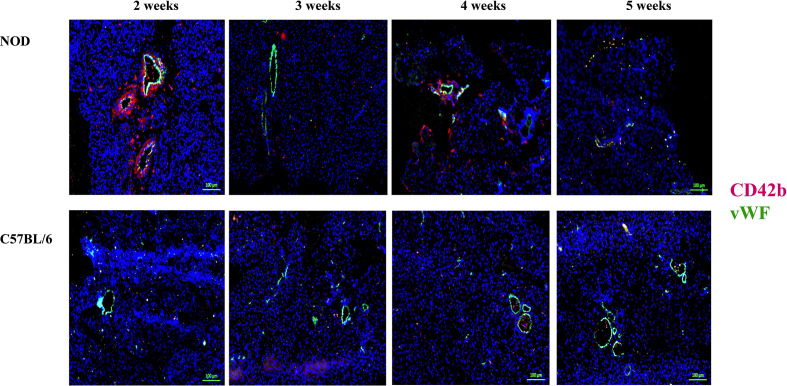
Post-natal pancreas from NOD female mice exhibit non-physiologica CD42b and vWF co-localization inside the pancreas as early as 2 weeks of age. Serial sections from formalin-fixed paraffin-embedded pancreata of 2-5 week old female NOD and C57BL/6 mice were stained with fluorochrome-tagged antibodies to CD42b and von Willebrand factor (vWF). Immunofluorescence shown is representative of outcomes observed in at least three individual mice of each strain. Bar at the bottom of each image represents the length of 100 μm.

**Figure 5:**
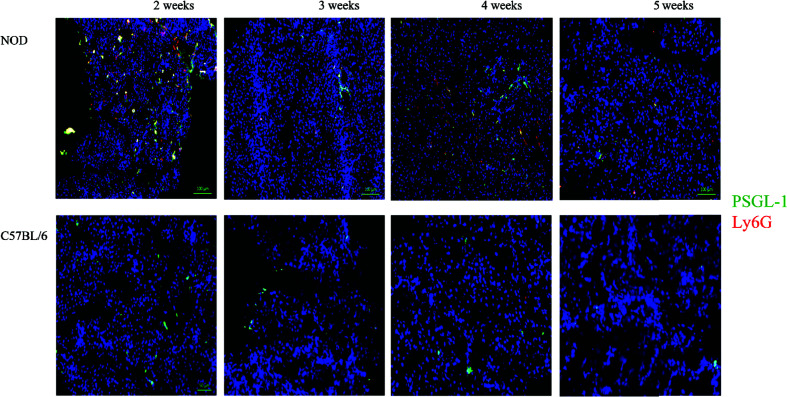
Generalized distribution PSGL-1 and Ly6G in the non-endocrine tissue of NOD, but not C57BL/6 pancreata at 2 weeks of age. Serial sections from formalin-fixed paraffin-embedded pancreata of 3-5 week old female NOD and C57BL/6 mice were stained with fluorochrome-tagged antibodies to PSGL-1 and Ly6G. Immunofluorescence shown is representative of outcomes observed in at least three individual mice of each strain. Bar at the bottom of each image represents the length of 100 μm.

During the co-staining of the pancreata serial sections with a number of different antibodies as an exploratory approach to identify potential etiopathology for the accumulation of neutrophils, we observed a remarkable co-accumulation of CD42b and vWF in what appears to be largely extra/peri-vascular localization (**Figure 4**). CD42b was localized around what appear to be ductal/vascular elements. This observation was consistent in all serial sections of 2 week old NOD pancreata only. Instead, only vWF was detectable, and in a predictable physiologic distribution, in serial sections from pancreata of 3–5 week old NOD mice. There was no absolute presence or absence of CD42b among all sections imaged; some sections exhibited CD42b while others did not (**Figure 4**). We did not observe significant CD42b-vWF presence in any of the serial sections from age- and sex-matched C57BL/6 mice although there were some sections that exhibited detectable CD42b-vWF (not shown). The presence of CD42b and vWF in a manner that appears to be extra/peri-vascular suggested that a substantial anomaly is occuring in the vascularization (61–66) of the non-endocrine tissue of NOD pancreata, at least in mice at 2 weeks of age. We proposed that any vascular anomaly would be reflected in an upregulation of P-selectin on the endothelium of these blood vessels. We also proposed that neutrophils would bind to P-selectin *via* the P-selectin glycoprotein ligand-1 (PSGL-1) (67–74) and that CD42b : von Willebrand factor would co-localize on platelets, whose accumulation on damaged endothelia would be expected to be both a response to, as well as a trigger of neutrophil arrest and margination inside the pancreas. Indeed, we observed a distinct overlapping of Ly6G and PSGL-1 in all the serial sections of 2 week old NOD pancreata (**Figure 5**) but none in the sections from pancreata of NOD mice of other ages. We did not observe any such co-localization in the serial sections of the pancreata of age-matched female C57BL/6 mice.

### Treatment of NOD Mice With Myeloperoxidase and Neutrophil Elastase Inhibitors, Alone or Together, Does Not Delay or Prevent the Onset of Diabetic Hyperglycemia

**Figure 6:**
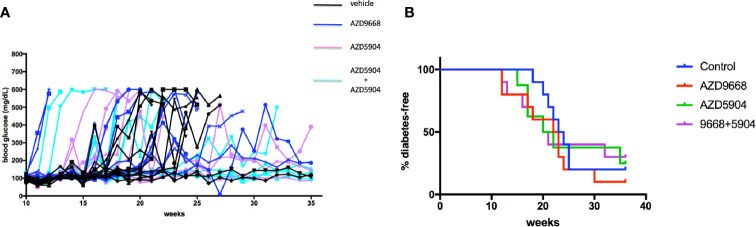
Diabetes incidence in NOD mice treated with AZD9668 alone or together with AZD5904. **(A)** Blood glucose concentrations in individual NOD female mice treated with AZD9668, AZD5904 alone, or in combination. Mice were allowed access to chow-formulated agents *ad libitum* beginning at 5 weeks of age. Blood glucose was measured once weekly until the time of diabetes confirmation. An n = 8–10 mice per treatment arm was monitored for the duration of this experiment. **(B)** Survival analysis. Log-rank test of the number of mice that became diabetic among all the treatment arms demonstrated that the study agents alone or in combination were ineffective in delaying or preventing the progression to diabetes compared to mice in the control treatment arm (*p* = non-significant).

The gathered immunofluorescence data compelled us to test if inhibition of key effectors of activated neutrophils could minimize, if not prevent, the potential pathologic effects of accumulating neutrophils inside the pancreas especially if neutrophils are critical bridges that determine the time at onset of activation of the adaptive arm of the immune system (i.e. the activation of islet-specific lymphocytes) in the NOD mouse strain. Indeed, others have shown that neutrophil antagonists can prevent the progression of T1D in NOD mice (23, 28, 33). Our approach was more directed as it targets only the major enzymes produced by neutrophils that underlie a significant aspect of localized tissue damage, namely myeloperoxidase and neutrophil elastase. We considered these enzymes to be important therapeutic targets given that they are present in abundance on NETs which underlie key mechanisms of neutrophil-mediated tissue damage (26, 49, 52, 59). Beginning at 3 weeks of age, NOD mice (n = 8–10 in each of the study agent arms) were fed *ad libitum* with a chow that was formulated with either AZD5904 [an inhibitor of myeloperoxidase (42)], AZD9668 [an inhibitor of neutrophil elastase (41)], or both. We established that the amount of chow consumed by the mice would result in a steady state level of each of these agents *in vivo* in line with a pharmacologic outcome comparable to that obtained in previous animal studies using these agents (41, 42). Mice were euthanized when diabetes was confirmed. In **Figure 6**, we show that neither agent alone or in combination was able to delay or prevent the progression of the disease to overt hyperglycemia compared to vehicle-treated mice. To ensure that the inability of the drugs to affect diabetes incidence was not due to inefficacious enzyme inhibition *in vivo*, potentially reflecting an inefficactious concentration inside the spleen and/or pancreas, we measured NE and MPO activity in pancreas and spleen homogenates in mice fed with control or drug-manufactured (AZD9668+AZD5904 together) diets for 14 days. In **Supplemental Figure 1**, we show that NE activity was lower in spleen and pancreas homogenates from control and combination drug-manufactured diets, reaching statistical significance in the spleen measurements, but not in the pancreatic measurements, although the mean in pancreas was substantially lower. Although we were unable to detect MPO inside the pancreata resected from the mice of the indicated ages, we infer MPO expression by detectable bioactivity as shown in **Supplemental Figure 2**. At the same time, in spite of repeated experiments, accounting for all possible technical shortfalls, we could not detect any statistically distinguishable differences in MPO activity in spleen or pancreatic homogenates from control diet and drug-manufactured diet-treated mice (**Supplemental Figure 2**). As an additional verification of drug activity *in vivo* to ensure that absence of an anti-inflammatory effect was not due to inadequate drug activity, we used a mouse model of acute lung injury to verify the activity of the inhibitors *in vivo* when administered *via* the food. Towards this objective, C57BL/6J female mice (n = 10 male and n = 10 female per treatment arm) were randomized to be fed control or drug-manufactured food (AZD5904+AZD9668 together) *ad libitum* for 14 days. On day 15, the mice in each treatment arm were randomized to be given an LPS solution (n = 5; 2.5 mg/kg body weight *Escherichia coli* LPS, serotype 055:B5; Sigma) or sterile saline (n = 5) *via* intratracheal instillation using a 20-gauge catheter. Mice were euthanized 18–22 h later. Bronchoalveolar lavage fluid (BALF) and lungs were collected. BALF was centrifuged and the supernatant used for measurement of total protein using the BCA Protein Assay Kit. Cell pellets were examined for the total number of leukocytes using a hemocytometer. MPO and NE activity were measured in the BALF leukocytes. We show the outcomes in **Supplemental Table 1** and confirm that the drugs are active in a setting of strong lung inflammation.

Although prevention of diabetes was not achieved using AZD5904 and AZD9668, it was potentially possible that these agents could have had an anti-insulitic effect and if this was evident, an additional, subtle effect could be achieved; selectively suppressing pancreatic β cell-reactive T-cells in a transient manner, even though this would be lost with the progression of autoimmunity towards overt hyperglycemia. Given that pancreas of NOD mice exhibits progressive insulitis as soon as 8–10 weeks of age, 7 week old female NOD mice were fed with no drug (n = 5) or the combination of AZD5904+AZD9668 (n = 5) for 2 weeks. Pancreata from euthanized mice at this time were cut into serial sections and stained with hematoxylin and eosin and histology was conducted by light microscopy. In **Supplemental Figure 3** we show pancreas histology representative of all the mice in the two treatment arms. Neutrophils are visible and evident by they are not frequent. There are no apparent differences in neutrophil numbers in and around the islets of the pancreata of mice between the two treatment arms. Insulitis is evident (as expected) and consists of a dense accumulation of lymphocytes. There are no significant differences in the quality of the insulitis in and around the islets of pancreata from mice from the two treatment arms.

## Discussion

A number of issues are raised by the data shown in this report. First, we confirm the accumulation of neutrophils as early as 2 weeks of age in the pancreas of the NOD mouse. Our observations together with earlier data (9, 10, 16, 23, 28, 33) should now leave no doubt that a significant tissue anomaly begins at or immediately after the time of birth in the pancreas of the NOD mouse strain that recruits neutrophils into the pancreas. The second issue raised by our data lies in the range of mechanisms that could account for the novel observation of significant concurrent accumulation of platelets and vWF peri- and extravascular of pancreatic vessels at 2 weeks of age, which is not observed at later time points. vWF binds to collagen on the exposed substrate of damaged endothelial cells (75–82) and acts as a binding site for CD42b on platelets. Such binding is known to occur under high shear stress due to rapid blood flow in narrow blood vessels (83–90). Moreover, vWF binds to platelets during pro-coagulant events (75–82). There are some reports indicating that vWF may be involved in the *de novo* formation of blood vessels (81, 91–94). Our data suggest that at 2 weeks of age, NOD mouse pancreas experiences some modification of blood vessel diameter either due to naturally occuring tissue modifications (differentiation, re-generation), or pathologic changes in the vascularization. Such changes would be expected to alter intrapancreatic shear flow and could account for the observations we show in this report (**Figures 4** and **5**). The concurrent co-localization of PSGL-1 and neutrophils around the blood vessels (**Figure 5**) suggests a non-physiologic event, very possibly pathologic, otherwise neutrophils would not be expected to be found inside the same anatomic region. It is currently unclear whether these events represent the outcome of a microthrombosis and/or endothelial damage. Although the co-localization of Ly6G with PSGL-1 suggests that PSGL-1 is on neutrophils, there is also the possibility that other myeloid cells have also been recruited since PSGL-1 can be expressed on myeloid cells (95, 96). That this observation was not made in any of the serial sections from pancreata of 2 week old C57BL/6 mice, indicates that these events are NOD strain-specific. As shown in **Figure 1**, there is a significant CD31 positivity in the NOD pancreas beginning at 2 weeks, an observation not evident in age- and sex-matched C57BL/6 mice. Although CD31 is often considered an endothelial cell protein (44–47), the staining pattern suggests that this represents another event. We propose that the CD31 positivity observed in the NOD pancreata represents aged neutrophils, activated neutrophils undergoing clearance or neutrophils in the process of transmigration through the post-natal pancreas endothelium (97–105). Further studies are warranted to clarify the actual process underlying this observation.

Whether the vascular immunopathology we observe at 2 weeks of age is associated with any aspect of T1D autoimmunity (e.g. whether it represents an event that licenses the activation of β cell-selective autoreactive lymphocytes) or whether it represents a fundamental anomaly in the peri-/post-natal development of the NOD pancreas is unknown. Twenty years ago, Finegood and colleagues reported that NOD mice as well as Biobreeding rats (a rat strain that spontaneously develops a progressive β cell-targeting autoimmune diabetes) exhibit a significant wave of β cell apoptosis in the pancreata of both rodents, peaking at 2 weeks of age (106). They reported this post-natal apoptotic event in the kidney and the nervous system and suggested that it is a physiologic developmental process. In the absence of autoreactive lymphocytes, such remodeling—even if it exposed damage-associated molecular patterns (DAMPs)—would not be expected to provoke anything other than a tissue-specific self-regulating and transient inflammation. On a background of autoreactive lymphocytes selective for pancreatic self-antigens, such a process would instead reveal autoantigens, activate pancreas-resident antigen presenting cells (macrophages and/or dendritic cells) (3–5, 7), and result in the activation of the autoreactive T- and B-lymphocytes. Diana and colleagues identified an anti-bacterial process as the basis for neutrophil accumulation at 2 weeks of age. When considered together with the findings of Finegood and colleagues as well as our novel observations involving potential platelet responses, we propose a model, that is not exclusive of any of the possible mechanisms underlying each individual findings (4, 7, 23, 28, 106). In this model, it is possible that, at least in the NOD mouse strain, gut leakage post- or peri-natally results in pathologic accumulation of bacteria that indirectly incite a wave of apoptosis as noted by Finegood. In the process, the vasculature could be damaged and an attempt at remodeling of the vasculature could be occurring, resulting in changes in blood vessel diameter (affecting shear flow) that would facilitate the recruitment of platelets at the sites of high shear in blood vessels as well as those exhibiting shear stress-dependent and/or independent endothelial damage. As these events unfold, DAMPs would be sensed by neutrophils which would accumulate inside the pancreatic tissue following arrest at P-selectin+ cell regions, extravasation, and activation. NETosis would be expected at this time, activating tissue resident antigen-presenting cells (3–5, 7). This model can also accommodate the known time at onset of the initiation of β cell-directed autoimmunity in NOD and Biobreeding rats (15 days of age) (107–110).

The third issue our data raise is the reason underlying the remarkable density of citrullinated H3 histones, largely in the non-endocrine cells, of NOD pancreas but not in age-matched C57BL/6 mice (**Figure 2**). While this marker is widely accepted to serve as a reliable surrogate of NETs, we temper this interpretation with some caution. First, even though the staining using the antibody to detect citrullinated histone H3 (Abcam ab5103) has been validated in >100 studies by others [representative studies referenced (10, 111–118)], and there is no comparable staining of C57BL/6 pancreas serial sections (**Figure 2**), it is possible that what is being observed is not necessarily NETosis, but a coordinated wave of chromatin modification exposing histone H3 to citrullination events inside the exocrine tissue. This modification has been associated with changes in gene expression including modulation of pluripotency in stem cells (119–123). If our observations in **Figure 2** are representative of chromatin modification and not formation of NETs, this would be a novel finding in the NOD mouse strain with possible consequences for individuals who are at high risk for T1D. At this time we can only speculate about the cause for such a potential chromatin modification especially in what appears to be almost all of the non-endocrine cells, and event that also co-incides with the time of maximum neutrophil accumulation inside the pancreas of NOD mice. The obvious limitation of our study is that we did not pursue further a determination as to whether the presence of citrullinated histone H3 co-incides with NETs. For this, we need to verify that these citrullinated histone H3 signals co-incide with NET DNA strands (detectable by SYTOX Green, for example) and at minimum MPO or NE. Although we did co-localize citrullinated histone H3 signals with NE or MPO in some serial sections from both NOD and C57BL/6 mice, other sections did not exhibit this co-localization and, furthermore, the variability of these events among the different age pancreata was too frequent to allow us to make any determinative interpretations (data not shown).

Even though NE and MPO would be expected to be attractive targets in order to delay the pathology inside the pancreas that predisposes to the peripheral autoimmune trigger, treatment of early age NOD mice with AZD9668 (NE inhibitor) or AZD5904 (MPO inhibitor), alone or together, was unable to delay the time at onset or prevent the progression towards overt hyperglycemia any better than control in NOD mice. It was formally possible that these agents, even though they did not have any obvious effect on insulitis, lymphocyte accumulation, and neutrophil frequency (**Supplemental Figure 3**), they might have conferred transient subtle effects on neutrophils that could mechanistically translate into modulation of the accumulation of beta cell-reactive lymphocytes (e.g. T-cells), antigen-presenting cells (e.g. dendritic cells and macrophages) resulting in a possible transient suppression of β cell-reactive T-cell proliferation/effector function. Although this study cannot establish this possibility, the data in **Supplemental Figure 3** do not appear to support such a possibility.

Neutrophils use an overlapping array of pathways to foster a pro-inflammatory state including the production of reactive oxygen species and cytotoxic immunokines. Our results indicate that those pathways are likely more relevant in the tissue turnover that results in the activation of autoreactive lymphocytes. In fact, a large body of evidence supports the importance of these pathways and mechanisms in the early stage of the disease, prior to the onset of insulitis (124–128).

## Data Availability Statement

The raw data supporting the conclusions of this article will be made available by the authors, without undue reservation.

## Ethics Statement

The animal study was reviewed and approved by Allegheny Health Network IACUC.

## Author Contributions

YG, BP, and CE conducted the experimental work and wrote as well as edited parts of the manuscript. MT critically reviewed the manuscript for important intellectual content and NG was responsible for the design, the interpretation, and the overall compilation, editing, completion, and submission of this manuscript. All authors contributed to the article and approved the submitted version.

## Funding

This work was supported in part by awards from the NIH (R21 TR001728-01) and the JDRF (SRA-2016-318-S-B) to NG.

## Conflict of Interest

The authors declare that the research was conducted in the absence of any commercial or financial relationships that could be construed as a potential conflict of interest.

## Supplementary Material

Supplementary Figure 1 NE activity in spleen and pancreas homogenates from NOD mice treated with AZD9668+AZD5904-manufactured chow. Enzyme activity is presented as the mean of units of activity/mg protein among a minimum of five assay wells. The difference in the activity in spleen between control and drug-manufactured diet-treated mice is statistically significant by two-tailed Student’s t-test (p = 0.019). The data are representative of outcomes using supernatants from pancreata from five different control and drug-treated NOD mice.

Supplementary Figure 2 MPO activity in spleen and pancreas homogenates from NOD mice treated with AZD9668+AZD5904-manufactured chow. Enzyme activity is presented as the mean of units of activity/mg protein among a minimum of five assay wells. The difference in the activity in spleen between control and drug-manufactured diet-treated mice is statistically significant by two-tailed Student’s t-test (p = 0.019). The data are representative of outcomes using supernatants from pancreata from five different control and drug-treated NOD mice.

Supplementary Figure 3 Insulitis in NOD mice treated with AZD9668+AZD5904. The panels on the left side show insulitis in pancreas from control mice (no drug treatment). The histology in the two panels is from two randomly selected sections and representative of insulitis in n = 5 NOD female mice at 9 weeks of age. The panels on the right side show insulitis in pancreas from AZD5904+AZD9668-treated mice. These two panels are from two randomly selected sections and representative of insulitis in n = 5 NOD female mice at 9 weeks of age treated with drugs. There are no apparent differences in the density of lymphocytes in the insulitis. Neutrophils are present, but are few in frequency and mostly randomly distributed inside the insulitis. Histology viewed at magnification ×20.

Supplementary Table 1 Efficacy of AZD5904 and AZD9668 in vivo in the suppression of MPO and NE activity as well as accumulated leukocytes in lung BALF. Enzyme activity is presented as the mean of units of activity/mg protein among a minimum of five assay wells from individual BALF leukocyte homogenates. Cell number is presented as the mean number of cells collected from the BALF of mice in each treatment arm, measured inside a Neubauer hemocytometer ×10^4^. The data in the table represent the outcomes in a total of 20 mice per treatment arm (n = 10 male and n = 10 female). Enzyme activity (MPO and NE) was measured in recovered BALF leukocytes, *in vitro*. Outcomes among treatment arms are statistically significant (p < 0.05, two-way ANOVA).
