# Supplementary figures and images for: Neutrophil-Associated Inflammatory Changes in the Pre-Diabetic Pancreas of Early-Age NOD Mice
Source: Front Endocrinol (Lausanne). 2021 Mar 10;12:565981. doi: 10.3389/fendo.2021.565981 (PMC7988208; doi:10.3389/fendo.2021.565981)

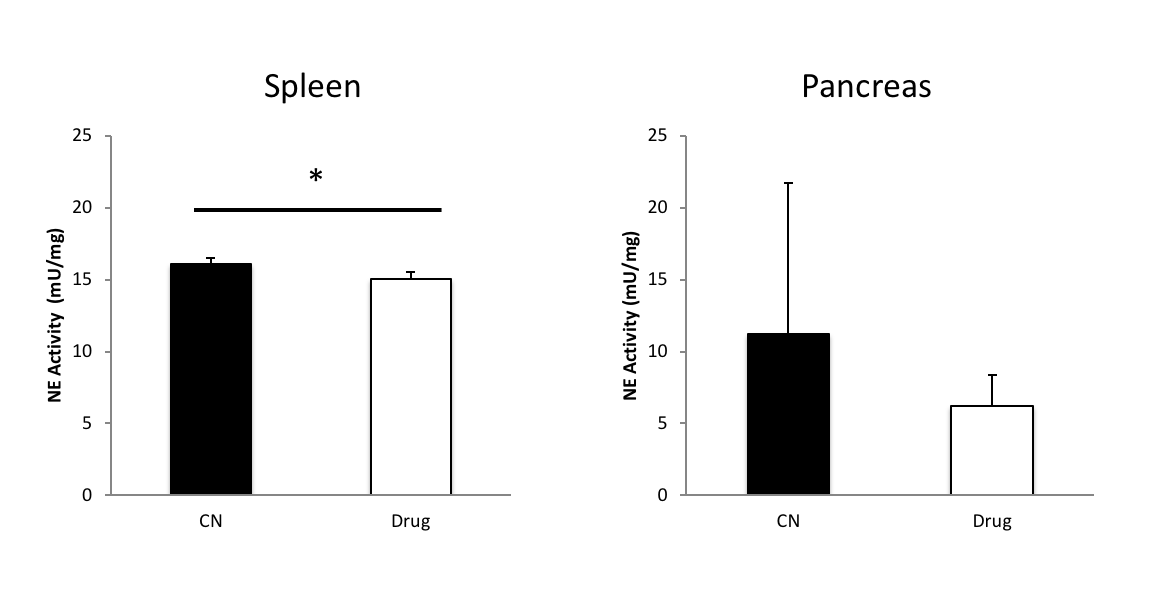

Supplement: Supplementary Figure 1 — NE activity in spleen and pancreas homogenates from NOD mice treated with AZD9668+AZD5904-manufactured chow. Enzyme activity is presented as the mean of units of activity/mg protein among a minimum of five assay wells. The difference in the activity in spleen between control and drug-manufactured diet-treated mice is statistically significant by two-tailed Student’s t-test (p = 0.019). The data are representative of outcomes using supernatants from pancreata from five different control and drug-treated NOD mice. [file Image_1.tif]

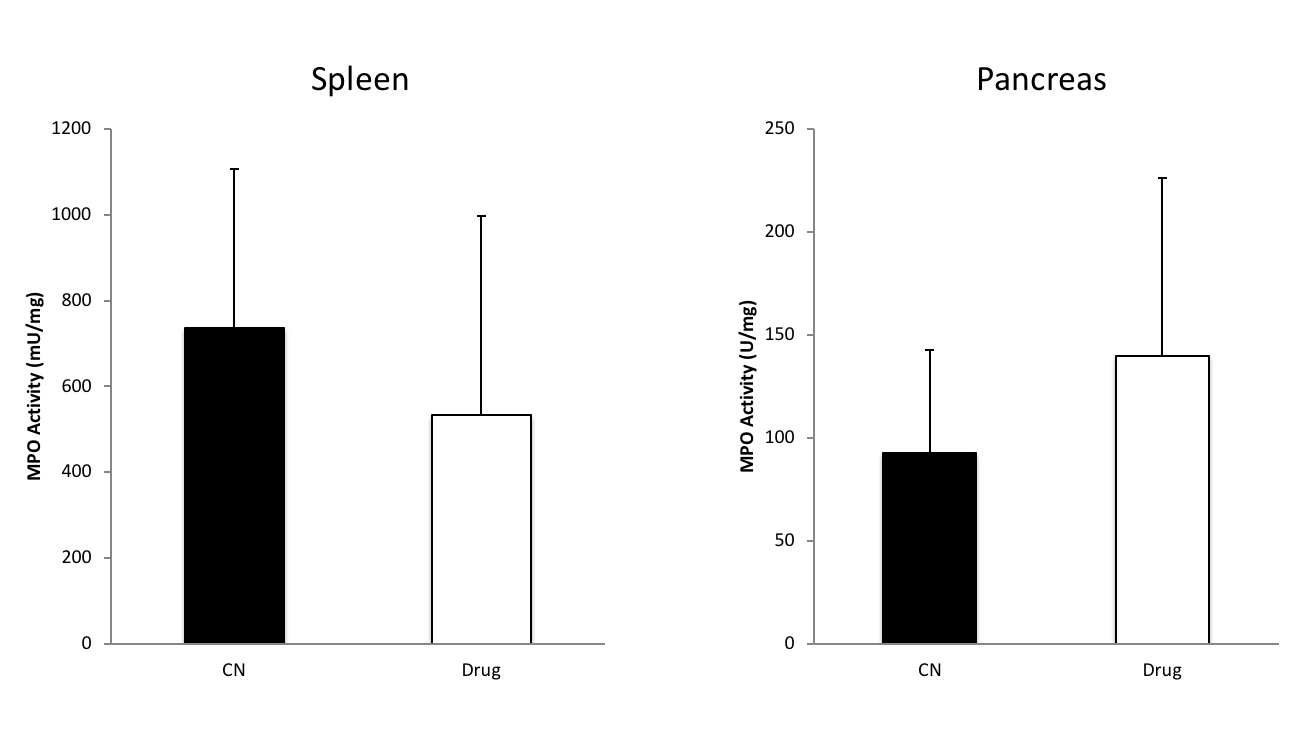

Supplement: Supplementary Figure 2 — MPO activity in spleen and pancreas homogenates from NOD mice treated with AZD9668+AZD5904-manufactured chow. Enzyme activity is presented as the mean of units of activity/mg protein among a minimum of five assay wells. The difference in the activity in spleen between control and drug-manufactured diet-treated mice is statistically significant by two-tailed Student’s t-test (p = 0.019). The data are representative of outcomes using supernatants from pancreata from five different control and drug-treated NOD mice. [file Image_2.tif]

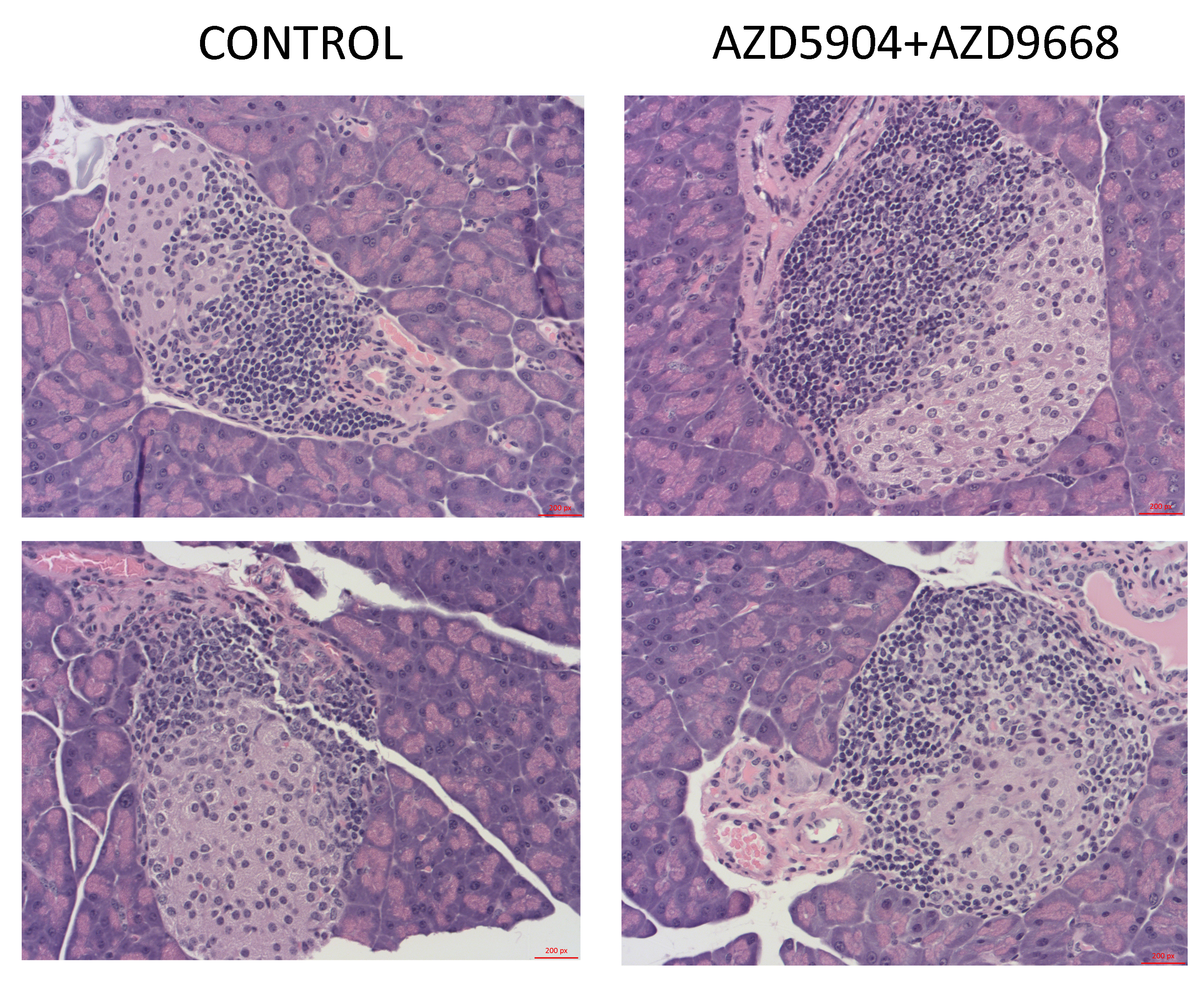

Supplement: Supplementary Figure 3 — Insulitis in NOD mice treated with AZD9668+AZD5904. The panels on the left side show insulitis in pancreas from control mice (no drug treatment). The histology in the two panels is from two randomly selected sections and representative of insulitis in n = 5 NOD female mice at 9 weeks of age. The panels on the right side show insulitis in pancreas from AZD5904+AZD9668-treated mice. These two panels are from two randomly selected sections and representative of insulitis in n = 5 NOD female mice at 9 weeks of age treated with drugs. There are no apparent differences in the density of lymphocytes in the insulitis. Neutrophils are present, but are few in frequency and mostly randomly distributed inside the insulitis. Histology viewed at magnification ×20. [file Image_3.jpeg]
