# Supplementary material for: Neutrophil-Associated Inflammatory Changes in the Pre-Diabetic Pancreas of Early-Age NOD Mice
Source: Front Endocrinol (Lausanne). 2021 Mar 10;12:565981. doi: 10.3389/fendo.2021.565981 (PMC7988208; doi:10.3389/fendo.2021.565981)
Supplement: Supplementary Table 1 — Efficacy of AZD5904 and AZD9668 in vivo in the suppression of MPO and NE activity as well as accumulated leukocytes in lung BALF. Enzyme activity is presented as the mean of units of activity/mg protein among a minimum of five assay wells from individual BALF leukocyte homogenates. Cell number is presented as the mean number of cells collected from the BALF of mice in each treatment arm, measured inside a Neubauer hemocytometer ×104. The data in the table represent the outcomes in a total of 20 mice per treatment arm (n = 10 male and n = 10 female). Enzyme activity (MPO and NE) was measured in recovered BALF leukocytes, in vitro. Outcomes among treatment arms are statistically significant (p < 0.05, two-way ANOVA). [file Table_1.docx]

**Supplemental Table 1**

| **Measurement** | **Treatment condition in vivo** | **Outcome** |
| --- | --- | --- |
|  |  |  |
| MPO Activity in vitro (U/mg protein) | Control (n=20) | 22 +/- 12 |
|  | AZD5904+AZD9668 (n=20) | 16 +/- 9 |
|  | LPS (n=20) | 147 +/- 36 |
|  | LPS+AZD5904+AZD9668 (n=20) | 48 +/- 14 |
|  |  |  |
| NE Activity in vitro (U/mg protein) | Control (n=20) | undetectable |
|  | AZD5904+AZD9668 (n=20) | undetectable |
|  | LPS (n=20) | 0.389 +/- 0.03 |
|  | LPS+AZD5904+AZD9668 (n=20) | 0.187 +/- 0.07 |
|  |  |  |
| Leukocyte recovery (x 10^4^/mL BALF) | Control (n=20) | 5 +/- 1 |
|  | AZD5904+AZD9668 (n=20) | 4.8 +/- 1 |
|  | LPS (n=20) | 1297 +/- 118 |
|  | LPS+AZD5904+AZD9668 (n=20) | 152 +/- 71 |
|  |  |  |
